# A text mining approach for CSR communication: an explorative analysis of energy firms on Twitter in the post-pandemic era

**DOI:** 10.1007/s43039-022-00050-3

**Published:** 2022-03-14

**Authors:** Rocco Mazza, Emma Zavarrone, Mirko Olivieri, Daniela Corsaro

**Affiliations:** 1 Università Degli Studi Di Napoli Federico II, Naples, Italy; 2 IULM University, Naples, Italy

**Keywords:** CSR communication, Energy firms, Text mining, Topic model, Twitter

## Abstract

The rapid diffusion of the Covid-19 worldwide has accelerated the need for companies to address the sustainability issue at different levels, as nowadays the attention of stakeholders with respect to this theme has grown considerably. As a result, companies had to set up CSR communication strategies to build and strengthen their legitimacy and reputation. Among the communication channels to convey messages of firms’ CSR initiatives, social media are becoming increasingly important and, particularly, Twitter is the social media platform where more CSR-related content is generated. By adopting the theoretical lens of constitutive communication of organization, the aim of this paper is to investigate with a textual approach how the CSR communication in the energy sector has evolved in the post Covid-19 scenario. Specifically, our attention will be focus on: (1) the exploratory analysis based on the hashtags; (2) the identification of CSR communication topics and (3) the proposal of topics network in order to discover subgroups of topics. Findings of this research show that the CSR communication on Twitter has undergone changes compared to the pre Covid-19 era. Particularly, we identified 11 CSR related-topics which, as the proposed topic network demonstrates, are interconnected. On the one hand, our results corroborate previous research regarding some CSR-related issues; on the other hand, we identified some topics such as safety, people and work which have exploded in Twitter conversations in the post Covid-19 scenario. Finally, this study provides managerial implications for professionals dealing with CSR communication, digital communication and social media marketing activities.

## Introduction

Since Bowen, in 1953, defined Corporate Social Responsibility (CSR) as a set of obligations of business men to pursue policies, make decisions and follow desirable courses of action in terms of the objectives and values of our society, this concept has evolved profoundly. As early as 1999, Carroll defined CSR as an essential part of business languages and practices, consistent with what the public expected from the business community of that time. Today, business development calls for an understanding of the relevance of implementing CSR with the aim to achieve legitimacy among consumers (Williamson et al., 2006; Cornelissen & Lancioni, 2008). Especially in the post-pandemic era, the topic of CSR has become crucial as a growing need to address sustainability at the economic, environmental and social pillars has been accelerated by the rapid spread of the Covid-19 (Pelikánová et al., 2021). In this complex scenario, an effective CSR communication strategy has become “a vital issue in building and sustaining the legitimacy and reputation of a company in the eyes of stakeholders” (Galati et al., 2019, p. 857). Indeed, nowadays, a key challenge for firms include issues about how to communicate and where to communicate the CSR initiatives. In fact, while consumers want to know the good deeds of the companies they buy from, they also quickly become wary of them when CSR initiatives are communicated and promoted aggressively (Du et al., 2010). In other words, companies today recognize the importance not only to engage in CSR initiatives, but also to communicate these initiatives to their target.

Among the channels in which these CSR initiatives are communicated by companies, social media represent a virtual space where the CSR corporate agenda can be shared with users, who actively interact with corporate content (Colleoni, 2013). As a matter of fact, on social media platforms consumers can comment on the initiatives of companies (Lee et al., 2013) engaging in a two-way dialogue with the firms (Tench et al., 2014). Although the academic literature on the CSR communication topic is very broad and has recognized that CSR-related issues being more and more communicated through digital platforms (Invernizzi & Romenti, 2020), scholars notice that research on CSR conversations on social media is limited (Chu et al., 2020) and that few studies are conducted in management field to investigate the way in which firms use social media to communicate their responsible behaviour (Galati et al., 2019). Particularly, previous research revealed that Twitter is the social media in which more CSR-related content is generated (e.g., Cortado & Chalmeta, 2016), as this platform is useful for companies both to increase awareness of the CSR initiatives and to facilitate the sharing of this content with stakeholders, promoting their engagement (Etter, 2014).

However, to our knowledge, no study has considered the evolution of how companies are communicating the CSR on Twitter in the post Covid-19 scenario. For example, the research by Chae and Park (2018) analyzed the topic prevalence, the topic correlation and the topic evolution of CSR-related themes on Twitter in the pre-pandemic scenario, focusing on the period between 2013 and 2016. Moreover, CSR literature has traditionally adopted a philanthropic perspective and the role of CSR initiatives in achieving social media marketing and communication objectives remains unexplored (Ahmad et al., 2021).

Hence, by considering the theoretical lens of constitutive communication of organization (CCO), according to which organizations maintain themselves through communicative practices (Schoeneborn et al., 2014), the overall aim of this study is to investigate the evolution of the CSR communication of companies in the post Covid-19 era. However, while previous studies have analyzed CSR communication mostly from stakeholders’ perspectives (Kim & Ferguson, 2014), for example by examining how the CSR can make a contribution to the corporate reputation (Bortree, 2014; Lewis, 2003), this study adds value to the scientific debate by considering CSR communication with a formative role in business practices (Cooren, 2012; Craig, 1999). This approach would allow to understand this phenomenon as a complex one that develops in companies in continuous negotiation processes (Ashcraft et al., 2009). The CCO perspective would thus allow to better capture the complexity of communication practice on social media, the involvement of multiple actors in the ecosystem as well as the ongoing nature of conversation as socially constructed.

At empirical level we conducted an exploratory hashtag analysis and, by following previous studies (e.g., Chae & Park, 2018), we examined the topic prevalence and the topic correlation of CSR communication of companies on the same social platform. Indeed, the construction of a tweet on CSR issues may not be a trivial task, as it is not always clear what aspect of CSR is important to convey on the chosen social media platform. It may happen that what management considers important and conveys using hashtags does not always correspond exactly to the theme dealt with in the tweet. In this direction, we formulate the three following research questions:

*RQ1*: How do companies use hashtags in their CSR communication strategies on Twitter in the post-pandemic scenario?

*RQ2*: What are the CSR-related topics addressed by companies in the tweets?

*RQ3*: How do the identified topics interrelate with each other?

To achieve our research aim, we move under the great umbrella of a textual approach where a textual analysis on the hashtags, a Latent Dirichlet Allocation (LDA) and textual network analysis using a two mode network approach (Everett & Borgatti, 2013) have been applied. LDA allows us to explore the content of our textual corpus and extract the main topics on which the selected company orient their social communication.

This paper is structured as follows. In paragraph 2 a literature review on the CSR communication in the corporate communication and marketing academic debate is presented, with a focus on the CSR communication strategies on social media. The methodology is illustrated in paragraph 3. Findings of this study are presented in paragraph 4, and the discussion and conclusions, managerial implications and limitations conclude the paper.

## Theoretical background

The aim of the following paragraphs is to outline the theoretical background on which our research was conducted. Particularly, we reviewed the literature on the main theories related to the CSR communication with a focus on studies which adopted constructivist perspectives and, subsequently, we analyzed studies on the role of social media in CSR communication strategies, seeking to identify the benefits and disadvantages of the adoption of these channels by companies.

### CSR communication in the academic debate

The academic literature focused on the Corporate Social Responsibility (CSR) topic is very broad and has considered different theoretical and methodological perspectives. Particularly, among the most recognized authors in the marketing literature, Kotler and Lee (2005, p. 3) define CSR as “a commitment to improve community well-being through discretionary business practices and contributions of corporate resources”.

Marketing and corporate communication scholars agree that CSR must be recognized as a key element of the strategic approach to communication (Nielsen & Thomsen, 2009), considering that organizations, by implementing this type of approach, are able to create a solid corporate identity, build a favorable reputation and establish profitable relationships with stakeholders (Cornelissen, 2004). However, CSR is still a confusing term about its meaning and nuances. On the one hand, according to Matten and Moon (2008), organizations define the CSR and its practices with different declinations depending on the social and cultural context in which they operate. On the other hand, as Cornelissen (2010) points out, scholars have attempted to propose classifications based on CSR issues. As a matter of fact, Garriga and Melè (2004) raise a classification considering four CSR group of theories, which are (1) instrumental theories, that consider CSR as a means for profits’ purposes; (2) political theories, that focus on the relationships between organizations and society as a social power tool; (3) integrative theories, according to which the business is influenced by the social values; (4) ethical theories, that believe ethical values are the basis of the relationship between organizations and society.

In general, the stakeholder awareness of the organizations’ CSR initiatives is considered by scholars as a key prerequisite for companies in order to achieve the strategic objectives of CSR (Du et al., 2010), which will then have to address the CSR communication theme. In this regard, the academic literature is configuring CSR communication more and more as an integral part of corporate communication (Berens et al., 2007; Pollach et al., 2012), given the growing importance acquired and the consequent increase in efforts of companies to implement CSR actions (Elving et al., 2015). In this vein, the perspective of communicative constitution of organizations (CCO), according to which companies would be invoked and maintained in and through communicative practices (Cooren et al., 2011; Schoeneborn et al., 2014), emerges clearly in several CSR communication studies. Indeed, according to the CCO approach, the reality is communicatively constituted and, specifically, the communication takes place “in name of” or “on behalf of” the organization (Schoeneborn et al., 2014).

Regarding CSR research which adopted explicitly or implicitly constructivist perspectives such as the CCO theory, CSR communication represents a growing trend, so much so that several studies have shown that CSR initiatives, if carried out effectively, allow organizations to obtain positive effects both in the short and in the long-term period for the organization (Nielsen & Thomsen, 2009). Specifically, according to a study by Schoeneborn and Trittin (2013) on the topic of CSR in CCO perspective, “the potential impact of CSR communication becomes a matter of connectivity of CSR to other practices of organizational communication” (p. 2). Indeed, some forms of CSR discourse would become actions within corporate strategies and, consequently, CSR approaches cannot be understood as “greenwashing”. The study by Schoeneborn and Trittin (2013) also shows that by adopting a CCO perspective, CSR communication broadens the boundaries of the company and involves third parties in business practices. Indeed, due to the proliferation of digital platforms such as social media, the sustainable marketing ecosystem has become increasingly complex (Sharmin et al., 2021, p. 18)—more actors are involved in this ecosystem and social media platforms are used today “to co-design, co-manage and co-market sustainable marketing experiences” with a positive impact on businesses.

However, Holme (2010) highlights that the interpretative uncertainty of CSR initiatives is exploited by companies to present themselves as socially responsible and in the best possible way, often over-emphasizing their performance and creating false expectations in stakeholders’ perceptions. The consequence is the widespread dissatisfaction with companies that communicate CSR and the skepticism regarding the veracity of the content of the CSR messages. This phenomenon is accentuated more in virtual spaces and, as Colleoni (2013) points out, especially on social media such as Facebook and Twitter, where stakeholders no longer passively receive messages from organizations, but evaluate their content with increasing attention. According to Christensen et al. (2013), in fact, consumers believe that what is “propagated” by organizations, for example on social media, may not correspond to the concrete business activities. The motivation behind this excessive emphasis in the communication of CSR initiatives by companies is also due to a persistent social demand which pushes companies to justify their business practices as well as contribute to the well-being of society (Scherer & Palazzo, 2011; Vollero et al., 2016).

In this sense, the CSR communication in terms of corporate communication is used as a means to obtain a form of social legitimacy (Bachmann & Ingenhoff, 2016; Castelló & Galang, 2014). Considering legitimation as defined by Suchman (1995, p. 574), that is “a generalized perception or assumption that the actions of an entity are desirable, proper, or appropriate within some socially constructed system of norms, values, beliefs, and definitions”, this objective is what allows the company to avoid social rejection. In fact, obtaining legitimacy by aligning the conduct of the company with the expectations of the stakeholders is what guarantees its existence and prosperity (Dawkins, 2004). To this end, CSR communication is configured as a tool aimed at achieving strategic objectives hidden by the communication of CSR initiatives, including the attempt to influence the target, the political, economic and social context. For example, from the studies by Brammer et al. (2012) and Nielsen and Thomsen (2009), it emerges that this instrumental communication of CSR initiatives is still predominant, and the content of the CSR message is determined by the interests of the business.

Therefore, it is clear that CSR communication is a means for companies to try to obtain greater trust, credibility and acceptance with their target and society. However, as argued by several authors (Benavides-Velasco et al., 2014; Calabrese & Lancioni, 2008), this communication activity must be carried out with particular care and consistency, as any discrepancy between social expectations and CSR initiatives actually implemented could have serious consequences for the firms, such as the loss of approval and respect or the stakeholders disengagement, as well as damage to the corporate reputation (Murray & Vogel, 1997; Park and Levy 2014). Indeed, the risk for organizations is to present CSR initiatives as marketing campaigns or a simple self-promotional action and translate as a form of hypocrisy of communication practices with respect to what the organization actually does (Christensen et al., 2020). While, CSR communication should focus on the organizations effort to bring concrete benefits to the society, and not be adopted for public relations’ purposes (Boatright, 2000) especially on new digital spaces.

### CSR communication on social media

Among digital platforms, social media represent a key channel to engage customers (Alves et al., 2016) as firms can easily establish a dialogue with them (Constantinides, 2014). With the development of these digital platforms, the way in which companies communicate their CSR initiatives has also evolved over time (Galati et al., 2019). Compared to old media, in fact, social media allow users to respond to CSR messages through the so-called user-generated content (UGC), ensuring shared value (Tench et al., 2014). Nowadays, companies communicate their CSR activities mainly through social media, which have profoundly reshaped the CSR communication by placing a dialogue between the company and stakeholders at the center (Tench & Jones, 2015). Social media, indeed, are considered virtual spaces in which CSR communication has become interactive and dialogic (Cho et al., 2017), allowing companies to establish relationships with users by responding to criticisms and reducing skepticism about corporate CSR initiatives (Dunn & Harness, 2018). Consumers, in fact, are increasingly critical and the communication of CSR activities plays an important role with respect to consumption choices and the evaluation of both internal and external stakeholders (Lee & Shin, 2010; Luhmann & Theuvsen, 2016). In this regard, several scholars highlight a positive link between CSR communication and consumers’ purchase intentions (Auger et al., 2003; Mohr et al., 2001). For example, a study by Ali et al. (2015) shows that CSR communication on digital platforms positively influences consumers’ purchasing intentions and contributes to improving the corporate image. Furthermore, other scholars demonstrate that social media users perceive companies that communicate their CSR initiatives in detail as more reliable than companies that communicate these initiatives in generic terms, without dwelling on particular issues (Robinson & Eilert, 2018). For this reason, issues regarding ethical problems are increasingly communicated through social media by companies (Russo & Simeone, 2017).

Hence, from the firm perspective, scholars have shown that, among the advantages of social media for CSR communication there are: (1) a wide reach of stakeholders in reduced times and costs and (2) the possibility of receiving immediate feedbacks by generating positive dialogues in order to establish a better relationship with the target. Contrariwise, the challenges of the CSR communication on social media concern (1) the management of any negative users’ comments on these virtual platforms, including Twitter, Facebook, YouTube, and (2) to reduce the skepticism of stakeholders (Ali et al., 2015).

Particularly with regard to Twitter, authors such as Chae and Park (2018) investigated how CSR-related themes are communicated and identified the prevalent topics in CSR dialogues. The two scholars adopt a computational content analysis and note some topics that Twitter users frequently discuss, for example: company strategy, community charity, climate and energy-related issues, supply chain, brand marketing; while, the topics concerning leadership, trust, management, benefit, and public are treated less on this platform. However, a study by Kollat and Farache (2017) has shown that a user who does not perceive an issue such as “being green” as close may find the company’s direct approaches on Twitter too invasive. In this regard, the social networks analysis by Etter (2014) also highlighted that most companies still adopt an asymmetrical communication approach to convey their CSR messages, focusing on issues relating to the environment, climate change and philanthropy, rather than employee relations, human rights or governance. In general, according to Etter (2014), Twitter is a platform both to increase awareness of the companies’ CSR initiatives and to manage relationships through user engagement strategies, facilitating the sharing of CSR content with stakeholders.

To achieve these engagement aims, CSR communication strategies on social media should be organized around the topics relevant to stakeholders, rather than as marketing, advertising or messaging tools (Kent & Taylor, 2016), as the stakeholder reactions can depend on the topics of the CSR communication (Kucukusta et al., 2019). For example, Kucukusta et al. (2019) highlight that the absence of posts encouraging consumer integration into CSR initiatives causes users to question their involvement in decision making in relation to CSR issues and, therefore, engagement strategies should be implemented by companies. However, many companies choose not to adopt user engagement strategies because the content of their CSR communication may not include answers to the concerns of their stakeholders, i.e. the social media users, and, therefore, would fail to meet their expectations (Lane and Devin 2018). As a matter of fact, Illia et al. (2017) demonstrate that companies still privilege CSR dialogues totally directed and moderated by themselves.

In sum, previous research shows the content proposed by the companies in these dialogues plays a key role. For example, according to the study by Kucukusta et al. (2019), in the pre-pandemic era, posts related to environmental CSR reach lower levels of engagement; while posts which encourage CSR behavior reach the highest level of engagement, as consumers imagine they are contributing positively to society (Schmeltz, 2012). However, a gap emerged from the academic literature, as no study considered which CSR-related topics were communicated by companies in the post Covid-19 scenario.

## Methodology

The textual umbrella allows to analyse the CSR communication in twofold perspective: descriptive and probabilistic ones. The pre-processing steps are fundamental because they structure the textual content and allow statistical processing. The first step of the strategy used was to generate an encoding of the documents and the creation of a corpus. From this each text was tokenized, obtaining a set of distinct strings (tokens) separated by spaces and punctuation marks. All other alphanumeric characters and symbols have been eliminated. Next, we reduced the characters of all terms to lowercase, a so-called normalization operation. We have eliminated stopwords, words useful to compose a complete sentence but which, taken alone, do not give any information (e.g. prepositions and articles). These tokens are called empty words or stopwords because they do not give any additional information. From this point begins the processing phase of the textual data as a lexical feature through an approach to lemmatization called Part of Speech. In this step we introduce an innovative approach, related to an automatization of this phase of data pre-processing which consist in an annotation of language elements in the texts with a language-agnostic tool, the R package UDPipe (Straka et al. 2017). We trained our annotator directly in the R environment following the standard preprocessing steps. The use of this approach is very innovative because the classical textual analysis does not operate with the training for the pre-treatment. From this point the data has been structured and we proceed to transform it into a numerical value by creating a documents-terms matrix. This is possible by vectorizing the lexical content of each document and counting the occurrences of each lemma within the textual units. When these phases are closed we applied the Latent Dirichlet Allocation in order to identify through a probabilistic approach the CSR communication topics.

### Latent Dirichlet allocation

**Fig. 1:**
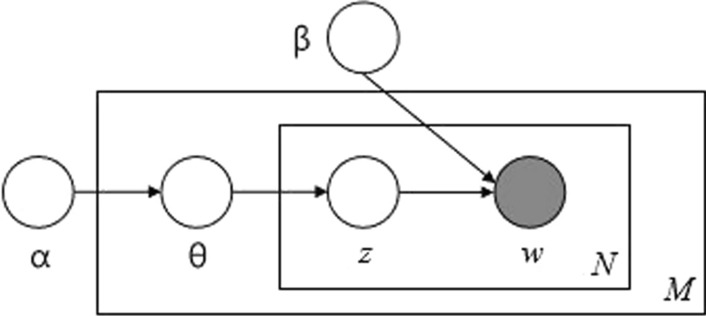
Graphical model representation of LDA (Blei et al., 2003)

When the aim is to identify the hidden themes that constitute the textual collections under analysis and the corpus is gathering a great amount of data, it becomes difficult to trace back the semantic structure constituting it. A topic model is a model that solves the issue and enables us to recognize a series of latent topics within a collection of texts: these topics are drawn out by the distribution of words. In the wide reference literature we rely on the following definition (e.g., Blei et al., 2003; Griffiths & Steyvers, 2002, 2003; 2004; Hofmann, 1999): topic models are probabilistic latent variable models of documents that exploit the correlations among the words and latent semantic themes. There is a large range of models proposed for the retracing and analysis of latent semantic structure and information within a corpus, and they all share the essential assumption that: “documents are mixtures of topics, where a topic is a probability distribution over words” (Steyvers and Griffiths 2007, p. 2). Starting from this point we can further define these models as generative models for documents: to generate a new text a topic is extracted, and subsequently a term from the distribution on the corresponding vocabulary; the process must be iterated along the entire length of the document. Among the great variety of probabilistic topic used to analyze the content of documents and the meaning of words (Blei et al., 2003; Griffiths & Steyvers, 2002, 2003; 2004; Hofmann, 1999), one worthy of attention is the Latent Dirichlet Allocation (from now LDA). The main reference to this peculiar model is certainly the work of Blei et al. (2003); moreover, the model has been extensively studied in Griffiths and Steyvers (2004), Blei and Lafferty (2007), Berry and Kogan (2010) and others. What follows in this section is an overview of the LDA topic model, largely based on the original authors’ work. At the start of the LDA we observe documents and words only: there are no evident topics, due to their being partly uncovered, therefore latent inside the structure of the document. The ultimate goal is to infer this latent structure of topics. The model carries out this task by recreating the documents in the corpus and by estimating the relative weight of the topic in the document and the word in the topic in an iterative way/fashion. The LDA is a Bayesian model and assumes that document and topic distributions can be described by a Dirichlet distribution (Blei et al., 2003). Arranging the process schematically (Fig. 1): from a Dir (α) Dirichlet distribution we carry out a random sampling which represents the distribution of the topics of a particular document; this topic distribution is *θ*; from *θ*, we select a particular topic **Z** based on the distribution; then from another Dirichlet distribution Dir (β), we select a random sample representing the word distribution of the topic **Z**, this distribution is *φ*, from *φ* we choose the word **w**. LDA has shown to work generally better than other models due to its potential to easily generalize new documents. In this model the data set acts as training data for the distribution of Dirichlet for topic-document distributions. The number of topics to extract from the corpus with this model is a parameter to be defined a priori. We need a methodological solution that allows us an automatic extraction of the topics. There are various methods for evaluating the model with the optimal number of topics (Blei and Lafferty 2009, Wallach et al. 2009; Buntine, 2009a, 2009b; Chang & Blei, 2009).

### The number of topics

In LDA topic modeling usually the number of topics to extract from the corpus is a parameter to be defined a priori, this can be a problem if an exploratory approach is adopted and an automatic extraction of the topics is carried out. The number of topics can affect the exclusivity of each topic and therefore can influence the tendency of semantic groups to overlap, as demonstrated in Cao et al. (2010). To avoid that an uncertain number could influence the results of this study, it was decided to adopt 2 different indicators. There are a variety of methods and algorithms for evaluating the model with the optimal number of topics (Blei and Lafferty 2009; Wallach et al. 2009; Buntine, 2009a, 2009b; Chang & Blei, 2009).

Cao et al. (2009) proposed another method used in this paper which provides an application of hierarchical Dirichlet process (HDP) to automatically learn the number of topics in the LDA model, based on the similarity between HDP and LDA in structure. Under this perspective, the best K of LDA is correlated with the distances between topics, it integrates the idea of clustering based on density. The method clustering on the various K models and identifies the one that minimizes the intra-cluster variance and maximizes that between clusters. In the end, he selects the best model through the following measure of perplexity:

$$ perplexity \left\{ {D_{test} } \right\} = exp\left\{ { - \frac{{\mathop \sum \nolimits_{{}}^{{}} }}{{}}} \right\} $$

The other measure used is taken from a work by Arun et al. (2010) in which the authors consider the LDA model as a matrix factorization mechanism:

$$ \mathop \sum \limits_{v = 1}^{W} M1\left( {t,v} \right) = \mathop \sum \limits_{d = 1}^{D} M2\left( {d,t} \right) \forall t = 1 \quad to \quad T $$

The measure proposed by the authors corresponds to the sum of the calculated values of the Kullback–Leibler divergence on two stochastic matrices M1 and M2:

$$ ProposedMeasure \left( {M_{1} , M_{2} } \right) = KL (C_{M1} ||C_{M2} ) + (C_{M2} ||C_{M1} ) $$

where C_M1_ is the distribution of singular values of Topic-Word matrix M1; C_M2_ is the distribution obtained by normalizing the vector L ∗ M2 (where L is 1 ∗ D vector of lengths of each document in the corpus and M2 is the Document Topic matrix).

### Data

Our methodological strategy is based on KPI selected from three areas of sustainability: social, environmental and economic. We preferred to analyze the communication of companies on Twitter, taking advantage of the mobilization on social media regarding environmental issues (Kirilenko and Stepchenkova 2014; Jang and Hart 2015; Cody et al. 2015; Veltri and Atanaova 2017; Segerberg & Bennett, 2011; Newman, 2017). We followed the profile tracking approach related to the study of companies’ communication on social media (Holsapple et al., 2018). In this regard, we have been part of a rich line of research in which researchers have been engaged for years in an interdisciplinary field that moves between business analytics and social media analysis (Ahuja & Shakeel, 2017; Birjali et al., 2017; Chang et al., 2017; Kale, 2016; Ketter, 2016; Kim et al., 2014; Öztürk & Ayvaz, 2018; Saif et al., 2016; Sitta et al., 2018; Wang et al., 2016).

We selected 5 energy company Twitter profiles based on sustainable KPI according to a study conducted by Statista (2021a). We have obtained from Twitter (through API) a sample of tweets among the leaders. In this pattern, we focused on the Twitter profiles with the highest number of followers and the highest number of posts published in Italy. The number of tweets for each company are reported in Table 1. At first glance, we can observe a different strategy of Twitter in the use of the language: it seems to be oriented to non-domestic areas for EnelGroup whilst the rest of the companies are focused on domestic areas.

**Table 1:** Selected companies for the research

Companies	Italian Tweets (%)	English Tweets (%)
EnelGroup	0.50	45.78
eni	20.67	17.44
ERGnow	28.62	10.63
snam	26.66	15.15
TernaSpA	23.55	11.00
Total (n)	8034	6653

## Results

The aim of the following paragraphs is to illustrate the results that emerged from our research conducted on CSR communication on Twitter in the post Covid-19 scenario.

### The exploratory hashtag analysis (RQ1)

The *RQ1* finds the answer in the explorative use of textual analysis through the comparisons among the selected hashtags both in English and in Italian. Following Zappavigna (2012, p. 1) the hashtag can be “as an emergent convention for labeling the topic of a micropost and a form of metadata incorporated into posts”. The social media literature shows a long series of studies on the descriptive use of the hashtag in the sustainability from the pioneer paper of Sutton et al. (2015) to the recent one of Yigitcanlar et al. (2021).

**Fig. 2:**
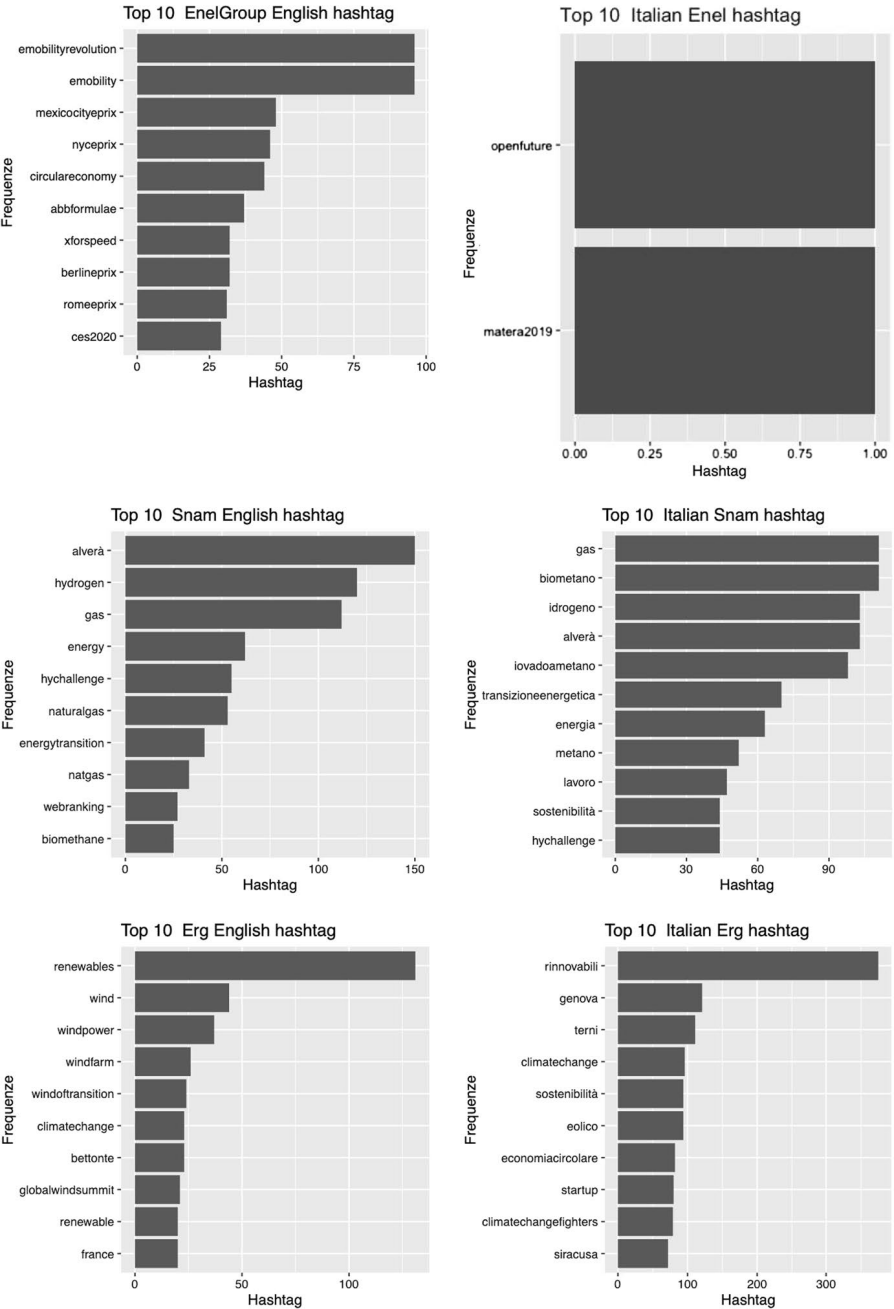

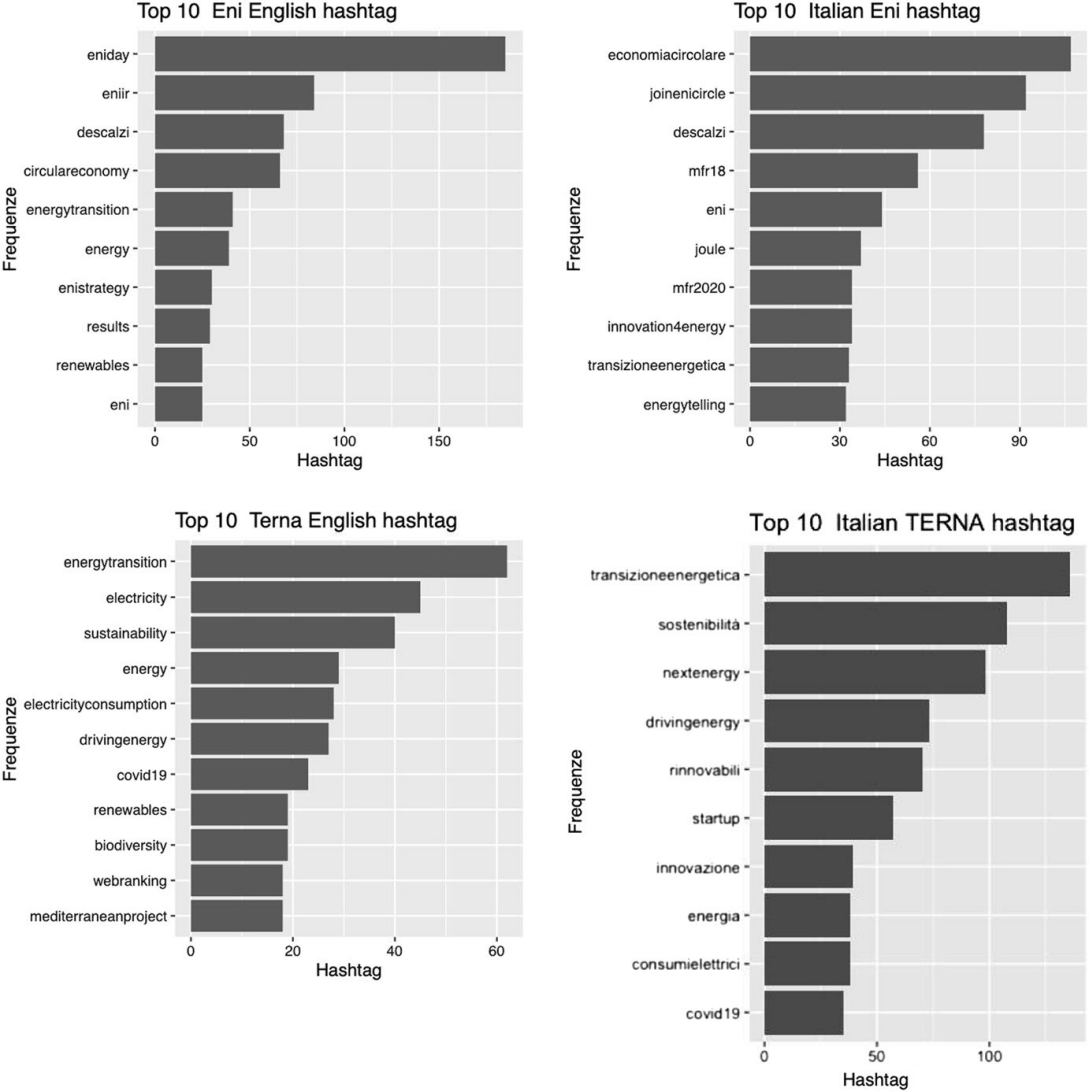
Comparison hashtag distribution (Top Ten)

So if we are interested in the identification of the themes, it is first of all important to verify which are the hashtags that synthesize the contents of the tweets. Since the CSR communication is dedicated to Italy we expect an identity of hashtags in both languages. This hypothesis, as we will see, is rejected because what emerges is a CSR communication not only distinct by type of hashtags but also by language. The simplest explanation is to be found in the plurality of audiences that CSR communication intends to engage. Figure 2 highlights these differences. It is reasonable to think that tweets follow a specific social media strategy for a country and so the generation of hashtag could be linked to specific strategy. It is important to discover that for Enel Group company the number of hashtags is negligible and the content of tweets belongs to customer area and disservices instead of CSR.

This result highlights the necessity of conducting the exploration of topics in a disjointed way by language. Therefore, in the next steps we will explore only the corpus composed of tweets in Italian.

### The CSR communication topics on Twitter *(RQ2)*

Due to the different strategic approach followed for non domestic areas, we have chosen to continue our analysis only on Italian tweets. The preprocessing reduces the corpus size to 7829 tweets and the number of types were 1314. The sampled Italian tweets related to the EnelGroup seem to contain only customer care dimensions and are very widespread during the time.

**Fig. 3:**
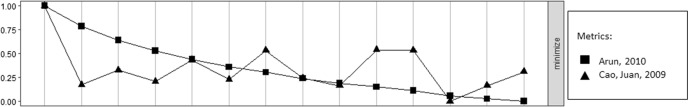
Selection of the optimal topics number

Figure 3 shows the calculation of the best model (Arun 2010; Cao and Juan 2009): the optimal number of topics is equal to the intersection between the two lines represented in the graph, at the points 13.

Table 2 shows the list of topics extracted and the most relevant lemmas for each topic. We describe the Italian content of these themes using the literature, however we can’t describe 2 topics because they represent the companies’ communication agenda.

**Table 2:** Topics and terms

Terms	Topic 1: Sustainability	Topic 2: Infrastructure	Topic 3: Research	Topic 4: Energy communication	Topic 6: Safety	Topic 7: Growth
1	sostenibilità	rete	progetto	energia	lavoro	milione
2	progetto	via	azienda	produzione	persona	investimento
3	valore	linea	startup	fonte	sicurezza	crescita
4	business	territorio	idea	emissione	iniziativa	miliardo
5	impresa	servizio	studente	sole24ore	ambiente	euro
6	modello	sviluppo	saperna	repowering	sfida	piano
7	innovazione	stazione	ambito	numero	competenza	risultato
8	territorio	realizzazione	ricerca	intervista	risorsa	domanda
9	comunicazione	intervento	vincitore	ruolo	storia	mese
10	relazione	comunità	soluzione	tempo	confronto	azione
Terms	Topic 8: Development	Topic 10: Gas	Topic 11: Future	Topic 12: Education	Topic 13: Energy transition	
1	sviluppo	gas	futuro	mondo	transizione	
2	innovazione	settore	anno	ragazzo	obiettivo	
3	strategia	accordo	evento	edizione	sistema	
4	centro	collaborazione	impiante	programma	decarbonizzazione	
5	opportunità	società	giorno	appuntamento	transizione energetica	
6	infrastruttura	mercato	novembre	scuola	percorso	
7	paese	biometano	esperienza	idel	ruolo	
8	tema	mobilità	mostro	vento	impegno	
9	attività	trasporto	momento	cambiamento	processo	
10	efficienza	metano	stand	team	Tecnologia	

### The topic network *(RQ3)*

**Fig. 4:**
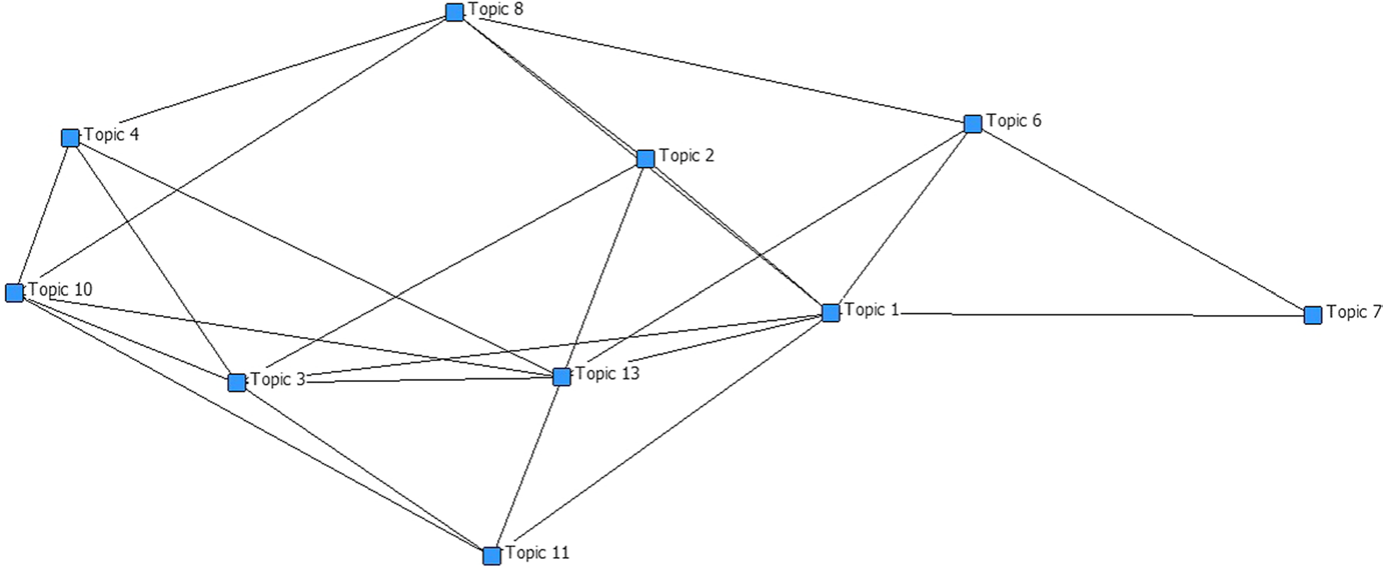
Topic network

After model application and topics extraction our strategy focused on the network analysis techniques to systematize, summarize and visualize the structure emerged to results of fitted model. We built a terms-topics adjacent matrix using the first 20 terms allocated for each topic. Subsequently we transformed this into topics-topics one-mode matrix, in this way we can study the relations between the extracted topics. These are based on the terms shared by several topics, for each shared word a link is generated between the topics. We plot a network from this matrix to visualize these relations. We described the network with centrality indicators (Faust 1997): degree, betweenness and closeness centrality. The degree of a node expresses the receptivity and popularity, the betweenness is the importance of the actor in terms of probability to be alone all the possible paths that connect the actors of the network and the closeness use the geodesic distance to identify the prestige of an actor, as the one that can most easily reach all the other players in the network. The topic networks in Fig. 4 express the links between the topics in relation to the sharing of words. The indices in Table 3 help to understand the relationships between topics.

**Table 3:** Centrality measures

	Degree	Closeness (norm.)	Betweenness (norm.)
Topic 1: Sustainability	7.000	0.706	0.169
Topic 2: Infrastructure	4.000	0.600	0.012
Topic 3: Research	10.000	0.706	0.158
Topic 4: Energy communication	6.000	0.571	0.012
Topic 6: Safety	4.000	0.571	0.045
Topic 7: Growth	2.000	0.444	0.000
Topic 8: Development	8.000	0.706	0.266
Topic 10: Gas	10.000	0.667	0.121
Topic 11: Future	4.000	0.571	0.006
Topic 13: Energy transition	10.000	0.667	0.083

Topic 3, topic 10 and topic 13 are the nodes with main degree, so they have the highest number of links. These themes are recurring topics in the communication, and have a shared vocabulary. The closeness measure denotes most important nodes, these are topic 1, topic 3 and topic 8. The betweenness helps to identify two sub-networks represented in network in Fig. 5, one links topic 1, topic 6, topic 7 and topic 8, this is the network of themes related to the dimension of sustainability and the other links topic 3, topic 4 and topic 10, this is the network related to the content communication.

## Discussion and conclusions

The overall aim of this research is to investigate how the CSR communication of companies evolved in the post-pandemic scenario. Specifically, we conducted an exploratory hashtag analysis (RQ1), identified the relevant CSR topics of the energy companies communication on Twitter (RQ2) and proposed a topic network to highlight how these topics interrelate (RQ3). In these different research steps, we have taken into consideration the theoretical lens of CCO according to which communication covers a formative role with respect to practices within organizations (Cooren, 2012; Schoeneborn & Trittin, 2013).

Generally, the contribution of this paper is twofold: on the one hand, we identify new dimensions of CSR communication emerged due to the Covid-19 pandemic and which, therefore, previous studies had not intercepted; on the other hand, through the analysis of the interrelation of the topics visible in the topic network, a systemic view of CSR communication on Twitter during the pandemic is provided. Moreover, although other studies which have analyzed the issue of CSR communication on digital platforms exist (Cho et al., 2017; Kucukusta et al., 2019), this is one of the first studies with a focus on the energy sector CSR communication in the post-pandemic scenario by adopting the CCO perspective. Indeed, this study contributes to the CCO research stream by highlighting the topics that are treated in the communication flows of the analyzed companies and that, according to the interpretation of Christensen et al. (2013), correspond to some actions that companies actually take. For example, as we will see in detail below, the topic of “Safety” emerges strongly as a new dimension of CSR communication and corresponds to a series of actions which companies are implementing to respond to the new needs of stakeholders. In fact, from the theoretical point of view, as highlighted in a recent study by Christensen et al. (2021), the so-called “talk-action dynamics” imply profound changes in companies and require time and resources to materialize. These dynamics can stimulate best practices within companies, specifically in the context of CSR, as “while we recognize that organizations emerge in an interaction between conversation and text, it is possible that this interaction is currently leaning towards the conversation side” (Christensen et al., 2021, 421).

Specifically, the first research question can be considered as a filter to test the possibility of analyzing CSR communications under a single lens. The role of exploratory analyses is now acquired in the demo-economic domains, we adopt this *modus operandi* in this study. The dual nature of CSR communication was highlighted by the use of exploratory analysis as well as implicitly inferring a distance between the terms used for hashtags and topics. Particularly, with this research we identified 11 CSR related-topics which, as the proposed topic network demonstrates, result interconnected. Topic 1, Topic 3 and Topic 13, which we have respectively indicated with labels Sustainability, Research and Energy Transition, represent the central hubs of our topic network map and, therefore, the focal points in the CSR dialogues of companies on Twitter. These results corroborate the study by Chae and Park (2018) on CSR communication on Twitter, according to which both the energy-related issues and the corporate strategy topic, that we have identified as dimensions of “Development” (Topic 8), characterize the CSR communication. In addition, from our study the theme of Research emerged as key element in the Twitter communication of energy companies. This shows that during the pandemic the issue of Research has also become crucial in terms of CSR. In fact, in the post-pandemic scenario, social media users assign greater value to the concreteness of the information and to the authoritativeness of the source (Statista, 2021b).

Our study also supports Etter’s social network analysis (2014), confirming that the topic of the Environment (Topic 6) is key in the discussions of CSR on Twitter. Specifically, from the proposed topic network emerged that the themes of the environment, the link with the territory and the country represent a set of topics addressed by energy companies today. Also, the topic of the Education, which we have identified as Topic 12, supports the recent research by Saxton et al. (2019), which states that education is a central area in social media CSR communication.

Regarding the new CSR dimension emerged from this study, we have noticed that the communication of CSR on Twitter has been strongly affected by the impact of the pandemic as issues such as “Safety” (Topic 6), with a focus on resources, people, work, which are emerging more and more clearly in the communication of companies on this digital platform. In fact, although Etter (2014) in his pre-pandemic research found that employee relations, human rights or governance were issues marginally addressed in CSR communication on Twitter, our investigation reveals that issues such as person, relationship, ideas are becoming central in the debate of CSR in post Covid-19 scenario. As a result of the pandemic, in fact, habits, expectations and priorities of consumers have radically evolved with a view to the general well-being of the person, that users also look for in relations with businesses.

These thematic clusters emerged from the analysis of communication flows on Twitter we conducted confirm that in a constructivist perspective communication becomes fundamental to dictate the agenda of the CSR activities that will be implemented within the organizations. Overall, research is showing that the topic of sustainability is key after the pandemic (Pelikánová et al., 2021) but its significance is becoming increasingly broad compared to previous studies (Chu et al., 2020). From this study, it emerged the CSR is today declined more widely than in the past within organizations and, consequently, this represents on the one hand a risk and on the other hand an opportunity for companies that communicate CSR on social media. In fact, social media are tools that during the pandemic have allowed companies to maintain relations with the external environment and to communicate CSR issues with a wide audience, reducing the degree of separation between businesses and users and making it easier to identify, reach and involve the stakeholders. For example, this has permitted companies to demonstrate a direct and tangible commitment in terms of CSR.

If on the one hand, therefore, it is convenient for companies to invest in CSR communication, “greenwashing communications” are often spread on social media, which tend to capitalize on the advantages of a business approach based on sustainability, trying to divert attention from one’s unethical conduct or not properly aligned with the principles of sustainability (Vollero, 2013). These practices are generating opposite effects in terms of the effectiveness of CSR communication and contribute to increasing the skepticism of social media users towards CSR communication. The risk is that in this scenario the benefits of CSR communication may also be reduced for companies that actually behave responsibly. Hence, increasingly, communication studies should take into account the entire sustainable marketing ecosystem on social media (Sharmin et al., 2021), in which companies operate, focusing on the perceptions of consumers and stakeholders in general.

## Managerial implications

This research provides several implications for managerial practices, particularly in relation to the management of the CSR communication on social media and Twitter. This paper, in fact, by identifying the key sustainability topics which are discussed on these digital platforms, provides guidelines for CSR professionals, social media managers and digital communication managers.

Specifically, our study highlights that some previously underestimated issues related to sustainability, such as safety and the link with the territory and the country, have become central in the post Covid-19 scenario. In this vein, communication professionals should take into account the evolution of the concept of sustainability when preparing CSR communication strategies for social media platforms.

Furthermore, our research shows that the theme of development and innovation (Topic 8) in relation to sustainability recurs more and more often in CSR dialogues on Twitter—for this reason, companies that decide to communicate their CSR initiatives on this platform should leverage on aspects concerning sustainable business innovation.

Finally, compared to the pre-Covid-19 era, the issue of the education, which was already widely communicated in the CSR communication strategies on social media (Saxton et al., 2019), has remained central: professionals, consequently, will have to continue to focus on this issue when communicating sustainability on Twitter.

## Limitations and future research

This exploratory study aims to investigate how the CSR communication evolved on Twitter in the post Covid-19 era and, as any research, it presents some limitations. First, this study was conducted considering a limited sample of 5 companies belonging to the same territorial context, namely Italy, and the same industry, i.e. the energy sector. Secondly, the research was conducted on only one social network, i.e. Twitter, and, consequently, does not consider the dynamics of the CSR communication evolution on other social media in the post Covid-19 days.

Thus, future research could therefore extend this perspective by investigating how CSR communication is evolving nowadays on other social media platforms and highlighting any differences both in terms of topics and interaction with users. For example, future studies could focus on the engagement and identify which types of content and which CSR topics generate the most engagement degree with Twitter and other social media users.
